# Programmed Cell Deaths and Potential Crosstalk With Blood–Brain Barrier Dysfunction After Hemorrhagic Stroke

**DOI:** 10.3389/fncel.2020.00068

**Published:** 2020-04-03

**Authors:** Yuanjian Fang, Shiqi Gao, Xiaoyu Wang, Yang Cao, Jianan Lu, Sheng Chen, Cameron Lenahan, John H. Zhang, Anwen Shao, Jianmin Zhang

**Affiliations:** ^1^Department of Neurosurgery, The Second Affiliated Hospital, School of Medicine, Zhejiang University, Hangzhou, China; ^2^Department of Physiology and Pharmacology, Loma Linda University School of Medicine, Loma Linda, CA, United States; ^3^Burrell College of Osteopathic Medicine, Las Cruces, NM, United States; ^4^Center for Neuroscience Research, Loma Linda University School of Medicine, Loma Linda, CA, United States; ^5^Department of Anesthesiology, Loma Linda University School of Medicine, Loma Linda, CA, United States; ^6^Department of Neurosurgery, Loma Linda University School of Medicine, Loma Linda, CA, United States; ^7^Brain Research Institute, Zhejiang University, Hangzhou, China; ^8^Collaborative Innovation Center for Brain Science, Zhejiang University, Hangzhou, China

**Keywords:** programmed cell death, necroptosis, ferroptosis, pyroptosis, blood–brain barrier dysfunction, crosstalk

## Abstract

Hemorrhagic stroke is a life-threatening neurological disease characterized by high mortality and morbidity. Various pathophysiological responses are initiated after blood enters the interstitial space of the brain, compressing the brain tissue and thus causing cell death. Recently, three new programmed cell deaths (PCDs), necroptosis, pyroptosis, and ferroptosis, were also found to be important contributors in the pathophysiology of hemorrhagic stroke. Additionally, blood–brain barrier (BBB) dysfunction plays a crucial role in the pathophysiology of hemorrhagic stroke. The primary insult following BBB dysfunction may disrupt the tight junctions (TJs), transporters, transcytosis, and leukocyte adhesion molecule expression, which may lead to brain edema, ionic homeostasis disruption, altered signaling, and immune infiltration, consequently causing neuronal cell death. This review article summarizes recent advances in our knowledge of the mechanisms regarding these new PCDs and reviews their contributions in hemorrhagic stroke and potential crosstalk in BBB dysfunction. Numerous studies revealed that necroptosis, pyroptosis, and ferroptosis participate in cell death after subarachnoid hemorrhage (SAH) and intracerebral hemorrhage (ICH). Endothelial dysfunction caused by these three PCDs may be the critical factor during BBB damage. Also, several signaling pathways were involved in PCDs and BBB dysfunction. These new PCDs (necroptosis, pyroptosis, ferroptosis), as well as BBB dysfunction, each play a critical role after hemorrhagic stroke. A better understanding of the interrelationship among them might provide us with better therapeutic targets for the treatment of hemorrhagic stroke.

## Introduction

Hemorrhagic stroke, including intracerebral hemorrhage (ICH) and subarachnoid hemorrhage (SAH), is an important public health concern with high morbidity and mortality worldwide (Scimemi, 2018; Fang et al., 2019). ICH and SAH have annual incidence rates of nearly 20 and 10 cases per 100,000, respectively, but with some regional variations (de Rooij et al., 2007; van Asch et al., 2010). Despite significant improvements in the prevention and clinical treatment of ICH and SAH, the global incidence, as well as related mortality and morbidity rates, has increased over the past several decades (Qureshi et al., 2009; Turan et al., 2016; Macdonald and Schweizer, 2017). As reported, the 5-year survival rate of patients with hemorrhagic stroke is less than 50%. Most survivors of hemorrhagic stroke have a reduced quality of life and may require more time and resources during hospitalization and rehabilitation (Gustavsson et al., 2011). Recently, numerous studies have focused on the pathological mechanisms after hemorrhagic stroke, with the intention of seeking potential therapeutic targets to guide treatment (Grysiewicz et al., 2008). However, because of the complexity of its mechanism and the limitations of translational studies, the benefits have been limited (Wilkinson et al., 2018). Hemorrhagic stroke occurs when a weakened vessel ruptures, allowing blood to traverse the broken blood–brain barrier (BBB) into the brain tissue, or fissures, which leads to a mass effect that increases the intracranial pressure and decreases the cerebral blood flow (Topkoru et al., 2017; Tao et al., 2019). Thus, the deficiency of ATP and increased blood toxic substances initiate a cascade of pathophysiological changes, such as depolarization, excitotoxicity, cell edema, inflammatory responses, oxidative stress, ionic homeostasis, and secondary BBB disruption (Zille et al., 2017; Fang et al., 2018; Marbacher et al., 2018; Shao A. et al., 2019; Shao Z. et al., 2019; Wan et al., 2019). These pathophysiological changes may lead to various forms of cell death, including apoptosis, necrosis, necroptosis, autosis, ferroptosis, pyroptosis, parthanatos, and cyclophilin D necrosis, which are also categorized under programmed cell deaths (PCDs; Fuchs and Steller, 2015; Sekerdag et al., 2018). Increasing efforts have been deployed to study these PCDs and their related pathways to discover potential therapeutic targets that will provide neuroprotection after hemorrhagic stroke (Leist and Jaattela, 2001).

Pathological changes in BBB function are widely implicated in the pathophysiological changes after hemorrhagic stroke (Daneman and Prat, 2015; Lublinsky et al., 2019; Ma et al., 2020). Recent studies have proposed that endothelial cells (ECs) do not work alone in the maintenance of the BBB and that astrocytes, pericytes, neurons, and microglia also contribute and are collectively known as the neurovascular unit (NVU). The death of these cells may directly or indirectly result in dysfunction of the BBB (Jiang et al., 2018). Additionally, BBB disruption may further aggravate brain edema, ionic homeostasis disruption, altered signaling, and immune infiltration, consequently causing cell death (Daneman and Prat, 2015).

Given the significant role of PCDs and BBB dysfunction in the pathophysiological mechanisms after hemorrhagic stroke, this review article focuses on the mechanism and recent studies of three PCDs (necroptosis, pyroptosis, and ferroptosis) after hemorrhagic stroke. In addition, we will also discuss, summarize, and hypothesize that the crosstalk between these PCDs and BBB dysfunction after hemorrhagic stroke may lead to the development of novel therapeutic approaches in the future.

## The Mechanism of Necroptosis

Necroptosis is a cell death similar to necrosis in terms of morphological features, such as being triggered by death ligands or intracellular stimuli and being executed in a caspase-independent manner (Liu et al., 2018). Morphologically, necroptosis is distinguished from apoptosis by the presence of clusters of dying cells, which may be indicated by early destruction of membrane integrity, cell and organelle swelling, cytoplasmic granulation, chromatin fragmentation, and cell lysis (Zhang et al., 2017). Ligands, such as tumor necrosis factor (TNF), TNF-related apoptosis-inducing ligand, and the intracellular stimuli, such as DNA-dependent activator of interferon regulatory factors (which may act as a cytoplasmic viral RNA sensor) and protein kinase R, can initiate necroptosis (Linkermann and Green, 2014). Furthermore, necroptosis can also be triggered by interferons and Toll-like receptor signaling (Zhang et al., 2017). The receptor-interacting kinase 3 (RIPK3) and its substrate, the pseudokinase mixed lineage kinase domain-like protein (MLKL), execute the core component of necroptosis without caspase participation (Zhang et al., 2009; Sun et al., 2012). Generally, there are several signaling pathways involved in necroptosis, including TNFα, TNF receptor (TNFR1)-related signaling pathway, TRAIL, and other factors associated with the apoptosis signaling pathway, as well as the RIPK3 mitochondrial reactive oxygen species (ROS) metabolic pathway and the zVAD-mediated PKC/mitogen-activated protein kinase (MAPK)/AP1-related signaling pathway (Jin and El-Deiry, 2006; Vanden Berghe et al., 2007; Wu et al., 2011; Liu et al., 2018).

**Figure 1:**
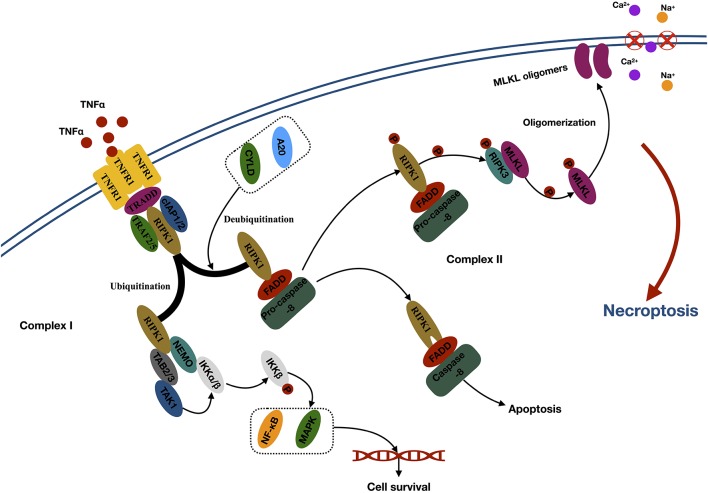
Mechanisms of necroptosis. Abbreviations: TNFα, tumor necrosis factor; TNFR1, tumor necrosis factor receptor type 1; TRADD, TNFα receptor-associated death domain; TRAF2, TNFR-associated factor 2; cIAP1, cellular inhibitor of apoptosis 1; RIPK1, receptor-interacting kinase 1; CYLD, cylindromatosis; FADD, Fas-associated death domain; RIPK3, receptor-interacting kinase 3; MLKL, mixed lineage kinase domain-like protein; NEMO, NF-κB essential modulator; TAB2/3, TAK1-binding 2/3; TAK1, TGF-β–activated kinase 1; IKKα/β, IκB kinase α/β; NF-κb, nuclear factor κB; MAPK, mitogen-activated protein kinase.

The first step of necroptosis is the formation of the necrosome (Zhang et al., 2017; Figure 1). Take the thoroughly studied TNFα–TNFR1-related signaling pathway as an example. TNF α activates TNFR1 through the extracellular domain and subsequently triggers the trimerization of TNFR1, which recruits several proteins to form complex I at the plasma membrane. Complex I comprised several components, such as TNFα receptor-associated death domain (interact with RIPK1), RIPK1 (core protein), TNFR-associated factor 2 (TRAF2), TRAF5, cellular inhibitor of apoptosis 1 (cIAP1), and cIAP2 (mediates the ubiquitination of RIPK1; Micheau and Tschopp, 2003). Ubiquitination of RIPK1 generates binding sites for TAB2/3 (transforming growth factor beta–activated kinase 1 (TAK1) and NEMO [the regulatory subunit of the IκB kinase (IKK) complex], which leads to further recruitment and activation of TAK1 and IKKα/β. Then, activated IKKα/β phosphorylated IκB and leads to nuclear factor κB release and MAPK activation, promoting cell survival (Bertrand et al., 2008; Hayden and Ghosh, 2014). Conversely, RIPK1 loses its default prosurvival function when it is deubiquitinated by the induction of deubiquitinases, such as cylindromatosis and A20 (Wertz et al., 2004; Moquin et al., 2013). Then, the deubiquitinated RIPK1 can bind to FAS-associated protein with a death domain and recruit procaspase 8 to form complex II within the cytoplasm. Apoptosis occurs when caspase 8 is activated. However, necroptosis occurs when caspase 8 is inhibited (Oberst et al., 2011). In the necrosome, RIPK1 can phosphorylate the RIPK3 to execute the following necroptosis (Zhang et al., 2009).

The second step of necroptosis is processing (Figure 1). The activated RIPK3 binds to and induces phosphorylation of cytoplasmic MLKL. Phosphorylation leads to the oligomerization of MLKL and membrane translocation of the MLKL oligomers, which disrupts the integrity and increases permeability of the membrane, therefore inducing internal flow of Ca^2+^ or Na^+^ ions, finally leading to cell death (Wang et al., 2014).

## Necroptosis in Hemorrhagic Stroke

Although the phenomenon of necroptosis has been extensively investigated in the pathophysiological mechanisms of other diseases, there are relatively few studies regarding necroptosis and hemorrhagic stroke (Table 1). It is known that necroptosis can be blocked by a small, specific molecular compound, known as necrostatin 1 (nec-1), by targeting and binding with RIPK1 (Galluzzi et al., 2017). Numerous studies have attempted to inhibit necroptosis by using nec-1 to reduce cell death and improve neurological function, thereby indirectly demonstrating the existence of necroptosis in the pathophysiological development after hemorrhagic stroke (Laird et al., 2008; King et al., 2014; Majmundar et al., 2016). Administration of nec-1 and the irreversible pan-caspase inhibitor, z-VAD, in astrocytic cells revealed that only nec-1 could significantly reduce hemin-induced astrocytic death, suggesting that caspase-independent necroptosis may mediate astrocytic death after hemorrhagic injury in an *in vitro* model. In addition, the proinflammatory effect of hemin relied on the enhanced astrocytic necroptosis after ICH (Laird et al., 2008). Meanwhile, nec-1 presented the capacity to reduce hematoma volume and neurovascular injury, while improving neurological outcomes after ICH in mice (King et al., 2014; Majmundar et al., 2016). However, they did not reveal detailed information pertaining to the cell types and mechanisms of necroptosis in their studies.

**Table 1:** Latest research of necroptosis in hemorrhagic stroke.

References	Stroke	Vitro/Vivo	Subjects	Related pathway	Conclusion
Laird et al. (2008)	ICH	Vitro	Mice primary astrocytes	/	Caspase-independent necroptosis mediates hemin toxicity in astrocytes
Chang et al. (2014)	ICH	Vivo	Mice	/	Nec-1 suppresses apoptosis and autophagy after ICH
King et al. (2014)	ICH	Vivo	Mice	/	Nec-1 reduces neurological injury after ICH
Su et al. (2015)	ICH	Vivo	Mice	RIPK1/RIPK3	Nec-1 ameliorates ICH-induced brain injury by inhibiting RIPK1/RIPK3 pathway after ICH
Xie et al. (2016)	SAH	Vitro/ Vivo	Rats/BV-2 microglial cells	SAP130/Mincle/Syk/IL-1β	Neuronal necroptosis product, SAP130, activates microglial Mincle and sequential inflammatory response after SAH
Majmundar et al. (2016)	ICH	Vivo	Mice	/	Nec-1 improved neurologic outcomes after ICH
Shen et al. (2017)	ICH	Vitro/Vivo	Rats/Primary neuron & microglia	TNFα/RIPK1/RIPK3/MLKL	Necroptosis is an important mechanism of neuron death in brain injury after ICH
Zille et al. (2017)	ICH	Vitro/Vivo	Mice and primary cultured neurons	RIPK1/RIPK3	Necroptosis involved in neuronal death mechanisms after ICH
Su et al. (2018)	ICH	Vitro	HT-22 cells	RIPK1/RIPK3	RIP1/RIP3 mediated hemin-induced neuron death, which can be reversed by nec-1
Chu et al. (2018)	ICH	Vitro/Vivo	Mice/Primary cultured neurons	IL-1R1/RIPK1/RIPK3	Inhibition of the interaction between IL-1R1 and the necrosome complex reduces neuron death and improves neurological functions after ICH
Chen et al. (2018)	SAH	Vivo	Rats	RIPK1/RIPK3/MLKL	Inhibition of RIPK3 attenuates early brain injury after SAH, possibly through alleviating necroptosis
Zhang et al. (2020)	ICH	Vitro/Vivo	Rats/AAV-293 cells	CHIP/RIPK1/RIPK3	E3 ligase CHIP inhibits neuron necroptosis and pathological inflammation following ICH
Yang et al. (2019)	SAH	Vivo	Rats	Nec-1 /CREB/BDNF	Inhibition of necroptosis by nec-1 rescues SAH-induced synaptic impairments in hippocampus
Lu et al. (2019)	ICH	Vitro/Vivo	Mice/HT-22 neuron cells	A20/RIPK1/RIPK3	Melatonin ameliorates microglial necroptosis by regulating A20 after ICH
Yuan et al. (2019)	SAH	Vitro/Vivo	Rats/Primary neurons & microglia	TNFα/RIPK1/RIPK3/MLKL	RIP3 induced neuron necroptosis involved in the pathological process after SAH
Chen et al. (2019)	SAH	Vivo	Rats	RIP1/RIP3/MLKL	Nec-1 attenuates brain swelling and BBB disruption and reduces necroptosis after SAH

It should be mentioned that nec-1 administration may also suppress the apoptotic and autophagic pathways after ICH and exert a neuroprotective effect (Chang et al., 2014). Necrostatin 1 treatment increased Bcl-2 expression and decreased cleaved caspase 3 levels, as well as the beclin 1/Bcl-2 ratio at 24 and 72 h after ICH (Chang et al., 2014). Thus, the following study attempts to identify the interaction between the key pathway in necroptosis (RIPK1/RIPK3) and nec-1. It has been found that RIPK1 and RIPK3 were significantly decreased, with reduced necrotic cell death (no detailed cell type was shown), under the treatment of nec-1 in mice after ICH, further suggesting that nec-1 inhibited necroptosis after ICH (Su et al., 2015).

The RIPK1/RIPK3 pathway was shown to play an important role in hemin-induced cell death (Zille et al., 2017; Su et al., 2018). RIPK1 and RIPK3 mRNA levels and phospho-RIPK1 increased after the primary cortical neuron had been treated with hemin (Zille et al., 2017). Necrostatin 1 or RIPK3 siRNA dramatically attenuated hemin-induced cell death and ROS accumulation in the HT-22 neuron cell line (Su et al., 2018). Furthermore, the neuronal necroptosis can be diminished if mutations are present at the serine kinase phosphorylation site of RIPK1, further supporting the significance of RIPK1 phosphorylation in necroptosis (Shen et al., 2017).

Meanwhile, the RIPK1/RIPK3-mediated necroptosis pathway was also involved in the alterations of pathophysiology after SAH (Chen et al., 2018, 2019; Yuan et al., 2019). The RIPK3 protein level increased in the rat brain and peaked at 24 h after SAH. Inhibition of RIPK3 by genetic or pharmacological treatment attenuated the brain injury in rats after SAH and neuronal necroptosis induced by oxygen hemoglobin (OxyHb; Chen et al., 2018, 2019; Yuan et al., 2019). Besides, pretreatment of nec-1 in rats after SAH demonstrated that nec-1 could also prevent BBB dysfunction by reducing the degradation of TJ proteins (occludin, claudin-5, and ZO-1), by increasing active matrix metalloproteinase 9 (MMP-9), and by reducing neuroinflammation *via* reduction of proinflammatory cytokines, such as interleukin 1β (IL-1β), IL-6, and TNFα after SAH (Chen et al., 2019). These findings seemingly indicated that nec-1 attenuated astrocytic and endothelial cell necroptosis. Additionally, it has been reported that the neuroprotective effect of nec-1 was also connected with another pathway. Necrostatin 1 rescues SAH-induced synaptic impairments and neuronal death in the hippocampus of rats *via* the cAMP-responsive element-binding proteins (CREB)/brain-derived neurotrophic factor (BDNF) pathway (Yang et al., 2019). cAMP-responsive element-binding protein and BDNF play an important role in synaptic plasticity, which contributes to memory processing (Seoane et al., 2011). Necrostatin 1 can reverse the decreased protein level of CREB and BDNF in rats after SAH (Yang et al., 2019).

Recently, several upstream regulators of necroptosis were found in hemorrhagic stroke (Shen et al., 2017; Chu et al., 2018). Necroptosis of primary cultured neurons could be induced by supernatant medium derived from microglia treated with OxyHb, but could be countered by a TNFα inhibitor, indicating that the TNFα–TNFR1-related signaling pathway participates in this process (Shen et al., 2017). Besides, hemin triggers neuronal necroptosis through promotion of IL-1 receptor 1 (IL-1R1) and RIPK complex formation. Inhibition of IL-1R1 can prevent necrosome and RIPK1/RIPK3 pathway activation, suggesting that the IL-1R1/RIPK1/RIPK3 pathway was also an important signaling pathway involved in necroptosis (Chu et al., 2018).

Downstream in the RIPK1/RIPK3 pathway, there are several regulators of necroptosis found in hemorrhagic stroke, such as carboxyl terminus of Hsp70-interacting protein (CHIP) and A20 (Lu et al., 2019; Zhang et al., 2020). CHIP is an E3 ligase that mediates ubiquitylation and negatively regulates the protein level of RIPK1 and RIPk3 (Seo et al., 2016). The expression of CHIP increased in the perihematomal region in rats after ICH. Overexpression of CHIP exerts neuroprotective effects by regulating the RIPK1/RIPK3 necroptosis pathway in neurons and therefore attenuating ICH-associated cerebral inflammation (Zhang et al., 2020). Additionally, the deubiquitylating enzyme, A20, was shown to inhibit RIP3 activity and reduce microglial necroptosis after ICH, *in vitro* and *in vivo*. Moreover, the A20 was identified as a novel target of melatonin, which upregulates A20 activity and suppresses necroptosis following ICH (Lu et al., 2019). On the other hand, it was found that the neuronal necroptosis product, SAP130, activates microglial macrophage-inducible C-type lectin (Mincle) and the sequential inflammatory response by mediating Mincle/Syk/IL-1b signaling in the ipsilateral hemisphere that had been subjected to SAH (Xie et al., 2017).

In conclusion, necroptosis is initiated after hemorrhagic stroke or hemin treatment in different cell types, such as astrocytes, neurons, and microglia. Inhibition of necroptosis by administration of nec-1 appears to be a potential target for the treatment of hemorrhagic stroke. However, the detailed mechanisms of necroptosis should be further investigated, not only *in vivo* but also *in vitro*, with particular emphasis regarding ECs due to the numerous studies demonstrating that inhibition of necroptosis can protect the BBB after hemorrhagic stroke.

## The Mechanism of Ferroptosis

**Figure 2:**
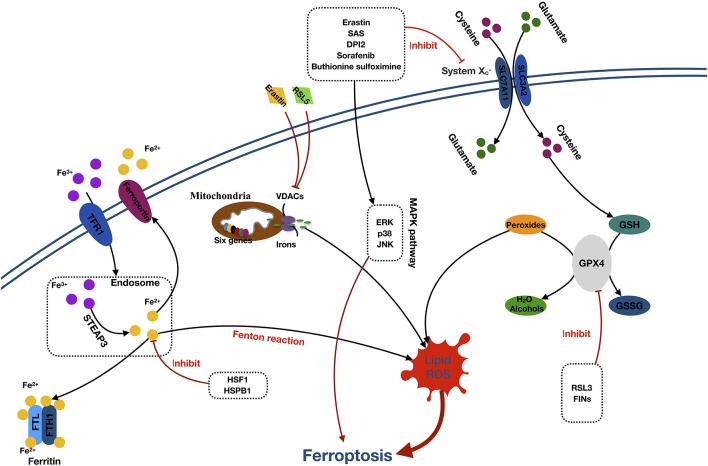
Mechanisms of ferroptosis. Abbreviations: TFR1, transferrin receptor 1; STEAP3, six-transmembrane epithelial antigen of prostate 3; FTL, ferritin light chain; FTH1, ferritin heavy chain 1; HSF1, heat shock factor 1; HSPB1, (HSF1)-heat-shock protein B1; RSL5, Ras selective lethal 5; VDACs, voltage-dependent anion channels; SAS, sulfasalazine; DPI2, diphenyleneiodonium 2; ERK, extracellular signal–regulated kinase; JNK, c-jun–N-terminal kinase; MAPK pathway, mitogen-activated protein kinase pathway; SLC7A11, solute carrier family 7 member 11; SLC3A2, solute carrier family 3 member 2; GSH, glutathione; GPX4, glutathione peroxidase 4; GSSG, oxidized glutathione; RL3, ribonuclease 1; FINS, fasting serum insulin.

Ferroptosis is a form of cell death characterized by the accumulation of intracellular iron and lipid ROS. The primary morphologic manifestations of ferroptosis include cell volume shrinkage and increased mitochondrial membrane density (Yu et al., 2017). The ferroptosis can be roughly divided into two segments: the core pathway of ferroptosis and iron metabolism (Xie et al., 2016; Figure 2).

The core component of ferroptosis includes lipid ROS accumulation due to the inhibition of System $X_{\text{c}}^{-}$ and glutathione peroxidase (GPX4). According to the different inhibition targets, the reducers can be divided into two classes. The first class includes erastin, sulfasalazine, DPI-sorafenib, and buthionine sulfoximine, which inhibits the depletion of System $X_{\text{c}}^{-}$ and glutathione (GSH; Xie et al., 2016). The second class comprised Ras-selective lethal 3 compound (RSL3), as well as DPI family members, which inactivate GPX4 (Yang et al., 2014). System $X_{\text{c}}^{-}$ is a membrane Na^+^-dependent cysteine–glutamate exchange transporter (with a ratio of 1:1) that is composed of a light-chain subunit (xCT, SLC7A11) and a heavy-chain subunit (CD98hc, SLC3A2). It is critical for maintaining redox homeostasis by reducing the intracellular cysteine that is required for the synthesis of GSH, a major antioxidant. When cells are cultured in glutamate, a substance causing neuronal hyperexcitability (Yang et al., 2019), the intracellular GSH content will decrease, and ferroptosis will be induced. Cells stimulated with class 1 ferroptosis inducers will usually have a significantly reduced GSH level (Yang and Stockwell, 2016). GSH peroxidase was a specific downstream site of GSH. GSH peroxidase uses two GSH molecules as electron donors to reduce H_2_O_2_, as well as other common small-molecule and complex lipid peroxides *via* decomposition of H_2_O_2_ into water or corresponding alcohols (Ursini et al., 1995). The class 2 ferroptosis inducers, such as RSL3 and FINs, directly inhibit GPX4 activity, contributing to the intracellular accumulation of lipid peroxides and subsequent ferroptosis (Yang et al., 2014).

The detailed role of iron in ferroptosis remains unclear. However, it was considered that the participation of iron is necessary for ferroptosis in oxidative reactions (Dixon et al., 2012; Gao et al., 2015). Free Fe^3+^ induces ferroptosis *via* importation into cells through the membrane protein transferrin receptor 1 and then stored in the endosome where Fe^3+^ is converted into Fe^2+^ by STEAP3. Subsequently, Fe^2+^ is transported by the divalent metal transporter 1 out of the endosome. The cytoplasmic Fe^2+^ is then transferred to three locations. Some is stored in ferritin, an iron storage protein complex, which includes a ferritin light chain and ferritin heavy chain 1. Some is transported out of the cell by the transmembrane protein, ferroportin. The rest is utilized in the Fenton reaction to create lipid ROS, which is an important source of ROS (Dixon and Stockwell, 2014; Xie et al., 2016; Wu et al., 2019). Moreover, erastin and RSL5 can alter the ion selectivity of the voltage-dependent anion channels, preventing the bidirectional movement of cations within the mitochondria, causing mitochondrial dysfunction and oxidant release (Yagoda et al., 2007; Yang and Stockwell, 2008).

Additionally, activation of the MAPK pathway and heat shock factor 1 (HSF1)–heat shock protein B1 (HSPB1) pathway also plays a significant role in ferroptosis. Inhibition of the MAPKs family, such as extracellular signal regulated kinase (ERK), p38, and c-Jun NH2-terminal kinase (JNK) can significantly attenuate the erastin-induced cell death, which indicates the potential role of MAPKs in ferroptosis (Yagoda et al., 2007; Yu et al., 2015). Furthermore, research has proven that HSF1 and HSPB1 can inhibit cell ferroptosis by decreasing the concentrations of iron and lipid ROS in cells (Sun et al., 2015).

## Ferroptosis in Hemorrhagic Stroke

Ferroptosis was first reported in cell death during tumor and embryonic development (Dixon et al., 2012; Jiang et al., 2015; Sun et al., 2015). However, few studies have reported this form of cell death in hemorrhagic stroke (Table 2). Morphologically, the mitochondria of ferroptotic neurons appeared shrunken, with decreased cytomembrane mitochondria at 3 and 6 days after ICH in mice. After several days, the swollen mitochondrial count appears to stabilize and is sustained until 28 days (Li et al., 2018). Interestingly, another study failed to find the shrunken mitochondria in primary neurons subjected to hemin stimulation (Zille et al., 2017). Regarding the molecular mechanism of ferroptosis, most studies attempt to confirm the presence of ferroptosis and the effects of ferroptosis inhibition *via* modulation of downstream pathways. To date, several ferroptosis inhibitors have been found. Accordingly, these inhibitors are primarily categorized as either antioxidants or iron chelators. The antioxidants include ferrostatin 1 (fer-1), liproxstatin 1 (lip-1), cycloheximide, N-acetylcysteine (NAC), Trolox, and U0126 (Dixon et al., 2012; Cao and Dixon, 2016; Zille et al., 2017), whereas the iron chelators include deferoxamine (DFO), ciclopirox (CPO), and dihydroxybenzoic acid (DHB; Okauchi et al., 2010; Karuppagounder et al., 2016).

**Table 2:** Latest research of ferroptosis in hemorrhagic stroke.

References	Stroke	Vitro/Vivo	Subjects	Related pathway	Conclusion
Karuppagounder et al. (2016)	ICH	Vitro	Primary cortical neurons/HT22 cells	HIF-PHD/ATF4	Iron chelators prevent glutamate or hemin-induced ferroptosis in neurons by targeting the HIF-PHDs/ATF4 pathway
Li et al. (2017)	ICH	Vitro/Vivo	Mice/Organotypic hippocampal slice cultures	/	Ferrostatin-1 inhibits neuronal death and provides neuroprotection by preventing lipid ROS accumulation and reducing GPX4 activity after ICH.
Zille et al. (2017)	ICH	Vitro/Vivo	Primary cortical neurons/mice	MAPK/ERK	Inhibition of ferroptosis abrogated hemoglobin-induced cell death *via* inhibition of MAPK/ERK1 pathway.
Karuppagounder et al. (2018)	ICH	Vitro/Vivo	Primary cortical neurons/mice		N-acetylcysteine functions as a neuroprotective therapy by preventing ferroptosis-generated toxic lipids.
Li et al. (2018)	ICH	Vivo	Mice	/	Ferroptosis existed in injured striatum during the acute phase of ICH.
Alim et al., 2019	ICH	Vitro/Vivo	Primary cortical neurons & HT22cells /Mice	GPX4 /TFAP2c/SP1	Pharmacological selenium inhibited GPX4-dependent ferroptotic death.

It was found that the neuron death and iron deposition, induced by hemoglobin in organotypic hippocampal slice cultures and primary cortical neurons, can be attenuated by administration of fer-1, or other ferroptosis inhibitors (Li et al., 2017; Zille et al., 2017). Ferrostatin 1 prevents Hb-induced GPX4 deficits and lipid ROS accumulation and attenuates the injury volume and neurological deficits after ICH in mice (Li et al., 2017). The phospho-ERK1/2 level was significantly elevated at 6 and 24 h after ICH in mice and blocked by U0126 (an inhibitor of MAPK), suggesting the involvement of the MAPK/ERK pathway in the regulation of ferroptosis (Zille et al., 2017). Regarding the downstream production of ferroptosis, cyclooxygenase 2 (COX-2), an enzyme encoded by the PTGS-2 gene, was found to be significantly increased within the first 3 days after ICH in mice and can be inhibited by fer-1 (Li et al., 2017). By using spectrometric analysis, the following study also proved that COX-dependent lipid species participate in the facilitation of ferroptosis (Karuppagounder et al., 2018).

N-acetylcysteine is a clinically approved cysteine prodrug, which modulates redox reactions by mediating activity of the $X_{\text{c}}^{-}$ transporter and the cysteine/glutamate ratio. Moreover, recent data suggest that NAC can inhibit ferroptosis *in vitro* and *in vivo* (Zille et al., 2017; Karuppagounder et al., 2018). N-acetylcysteine attenuates hemin-/hemoglobin-induced cell death in primary cortical neuronal cells and improved functional recovery after ICH in mice (Zille et al., 2017; Karuppagounder et al., 2018). In this study, arachidonate 5-lipoxygenase (ALOX5)–derived reactive lipid species was revealed to contribute significantly in hemin-induced ferroptosis. The NAC-mediated neuroprotective effect was executed by increasing GSH levels and subsequently neutralizing nuclear ALOX5-derived lipid species after ICH (Karuppagounder et al., 2018). Additionally, this study also found that the COX-2–derived species, PGE2 and NAC, could synergize to prevent hemin-induced ferroptosis *in vitro* and improve functional recovery after ICH (Karuppagounder et al., 2018). However, this result seems to be in contrast of the detrimental effect of COX-2 after ICH, as mentioned in the previous study (Li et al., 2017).

A recent study revealed that iron chelators (DFO, CPO, DHB) inhibit glutamate- or hemin-induced ferroptosis in neurons by targeting the hypoxia-inducible factor prolyl hydroxylase domain enzymes (HIF-PHDs), a family of iron-dependent enzymes necessary for ATF4-dependent prodeath transcription. However, this process was identified independently to the inhibition of the Fenton reaction (Karuppagounder et al., 2016). ATF4 is a leucine zipper transcription factor that is activated in the ferroptosis-induced transcriptional responses found in neurons and cancer cells. Neurons with a germline deletion of ATF4 had resistance to the homocysteic acid–induced ferroptosis (Lange et al., 2008; Karuppagounder et al., 2016; Chen et al., 2017). This evidence suggests that the ATF4-dependent pathway may have an important role in ferroptosis after ICH.

The GPX4 homeostasis is a critical component of ferroptotic cell death after ICH. However, little is known about the expression levels of GPX4 and other selenoproteins after ferroptotic initiation. The latest study found an increased expression of several selenium-containing antioxidant enzymes, including GPX4, thioredoxin reductase 1, GPX3, and selenoprotein P *in vivo* and *in vitro* (Alim et al., 2019). The neuron-specific expression of GPX4 after ICH functions as a protective factor to avoid neuronal loss from ferroptosis-induced oxidative damage. However, it seems to be an insufficient response in preventing cell death as a result of ferroptotic insults (Karuppagounder et al., 2018; Alim et al., 2019). Interestingly, this study found that selenium augments the transcriptional response involving GPX4 *via* coordinated activation of the transcription factors, TFAP2c and Sp1, to protect the neurons from ferroptotic death and improve functional recovery after ICH in mice (Alim et al., 2019).

It should be mentioned that there have not been any studies that investigate the role of ferroptosis in the pathophysiology after SAH. Our laboratory has found that lip-1 can reduce the characteristic shrunken mitochondria in ipsilateral cortical neurons after SAH in mice. Liproxstatin 1 participated in attenuation of active lipid ROS by reducing the production of malondialdehyde and 4-hydroxynonenal (4-HNE) and by increasing the level of GSH and the activity GPX. Furthermore, lip-1 downregulated acyl-CoA synthetase long-chain family member 4 and COX-2, but also reduced the activation of microglia and inflammatory response (data unpublished).

In conclusion, ferroptosis has a critical role in the cell death of neurons. However, there have not been any studies conducted that focus on other cell types. After hemorrhagic stroke, the iron metabolic disorders and ROS accumulation both existed in the endothelium, microglia, and astrocytes. In addition, the BBB dysfunction and inflammation were also shown to be associated with the ferroptosis inhibitors (Zhang Z. et al., 2018; Zhang Y. H. et al., 2018). Ferroptosis may also occur in these cells after hemorrhagic stroke. Further studies should expand their research beyond the role of ferroptosis in neuronal death.

## The Mechanism of Pyroptosis

**Figure 3:**
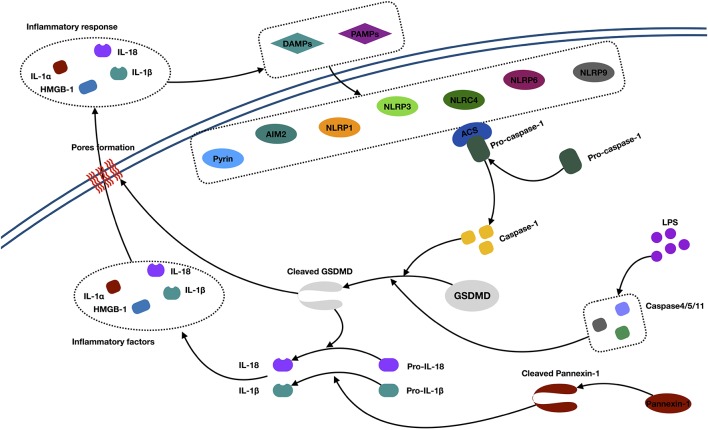
Mechanisms of pyroptosis. Abbreviations: IL-1α/1β/18, interleukin 1α/1β/18; DAMPs, danger-associated molecular patterns; PAMPs, pathogen-associated molecular patterns; AIM2, absent in melanoma 2; NLRP, (NOD)-like receptor protein; ACS, andacyl-CoA synthetase; GSDMD, Gasdermin D; HMGB1, high-mobility group box 1; LPS, lipopolysaccharide.

Pyroptosis is a proinflammatory form of cell death (Bergsbaken et al., 2009; Chen et al., 2018). Morphologically, pyroptotic cell death possesses features of both necrosis and apoptosis. The morphological changes include necrosis-like cell membrane rupture, pore formation, cellular swelling, proinflammatory intracellular content release, as well as apoptosis-like nuclear condensation and DNA fragmentation. In contrast to apoptosis, pyroptotic cells have an integral mitochondrion and “balloon-shaped” vesicle formation, but no cytochrome c release (Xu et al., 2018; Jia et al., 2019). Molecularly, pyroptosis is characteristic in gasdermin-mediated cell death (Shi et al., 2017). Normally, the gasdermin is activated by two caspase-dependent pathways, including canonical caspase 1 and the noncanonical caspase 4/5/11 pathway (Jia et al., 2019; Figure 3).

In the canonical caspase 1 pathway, inflammasomes (also called pyroptosomes) assemble in the cytosol after recognizing pathogen-associated molecular patterns and danger-associated molecular patterns released by dying cells and some proinflammatory cytokines (Lamkanfi and Dixit, 2014). Inflammasomes are multimeric protein complexes, which are composed of the nucleotide-binding oligomerization domain (NOD)-like receptor (NLR) family (NLRP1, NLRP3, NLRC4, NLRP6, and NLRP9), PYHIN protein families [absent in melanoma 2 (AIM2)], or pyrin proteins (Zheng et al., 2011; Sekerdag et al., 2018). Typically, an NLR consists of three parts: C-terminal leucine-rich repeats, which are responsible for ligand recognition and autoinhibition, a central NOD (NACHT), which activates the signaling complex, and an N-terminal caspase activation and recruitment domain (CARD) or pyrin domain (PYD), which mediates homotypic protein–protein interactions (Martinon et al., 2002; Aachoui et al., 2013). The AIM2 contains a DNA-binding HIN-200 domain and PYD. The pyrin protein consists of the PYD, a zinc finger domain (bBOX), a coiled coil domain, and/or a B30.2/SPRY domain (Jia et al., 2019). The signaling domains, such as the PYD and CARD, both recruit the apoptosis-associated speck-like protein containing a caspase recruitment domain (ASC). This subsequently activates pro–caspase 1 to generate active caspase 1 (Aachoui et al., 2013). Then, the activated caspase 1 cleaves the gasdermin D (GSDMD) and triggers oligomerization of the GSDMD-N domain in the cell, which finally forms the pores and releases cellular contents, such as IL-1α and HMGB1. Besides, the active caspase 1 is also responsible for processing and maturing IL-1β/18, which cause inflammation (Dinarello, 2011; Xu et al., 2018).

In the noncanonical caspase 4/5/11 pathway, caspase 4/5/11 was activated by a direct interaction with cytosolic lipopolysaccharide. Next, activated caspase 4/5/11 directly initiates the cleavage of GSDMD and triggers pyroptosis. Meanwhile, active caspase 4/5/11 can initiate pannexin 1 cleavage and K^+^ efflux, indirectly facilitating the release of mature IL-1β (Kayagaki et al., 2011; Cheng et al., 2017).

## Pyroptosis in Hemorrhagic Stroke

Pyroptosis, unlike other types of PCDs, cannot be detected morphologically. Thus, the existing research is limited to the molecular level (Table 3). It was shown that the NLRP3 protein is upregulated after ICH and SAH and peaked at 24 h, along with elevation of inflammatory factors, IL-1β and IL-18 (Chen et al., 2013; Ma et al., 2014; Feng et al., 2015; Dong et al., 2016). Meanwhile, the significant upregulation of caspase 1 was detected at 3 h and peaked at 24–72 h after ICH (Ma et al., 2014; Feng et al., 2015). Inhibition of caspase 1 by Ac-YVAD-CMK could significantly decrease brain injury presentation, as evidenced by improved neurological functions, and amelioration of brain edema after ICH (Wu et al., 2010; Lin et al., 2018). The neuroprotection was associated with decreased expression of IL-1β, JNK, and MMP-9 and inhibition of ZO-1 degradation (Wu et al., 2010). The Ac-YVAD-CMK administration reduced M1-type microglia polarization and increased the number of M2-type cells around the hematoma (Lin et al., 2018). This characteristic polarization of microglia was also reported to reduce the poststroke neuroinflammatory damage in an ischemic stroke study (Xu et al., 2019).

**Table 3:** Latest research of pyroptosis in hemorrhagic stroke.

References	Stroke	Vitro/Vivo	Subjects	Related pathway	Conclusion
Wu et al. (2010)	ICH	Vivo	Mice	JNK/caspase-1/Il-1β	Caspase-1 inhibitor, Ac-YVAD-CMK, reduced brain injury *via* downregulation of IL-1β, JNK, MMP-9, and inhibition of ZO-1 degradation after ICH
Chen et al. (2013)	SAH	Vivo	Rats	P2X7R/NLRP3/IL-1β(18)	P2X7R/NLRP3 axis inhibition reduces neuroinflammation after SAH
Ma et al. (2014)	ICH	Vivo	Mice	ROS/NLRP3/caspase-1/Il-1β	ICH activated the NLRP3 inflammasome and the following inflammatory response, which may be stimulated by mitochondrial ROS.
Dong et al. (2016)	SAH	Vivo	Mice	NLRP3/Il-1β	Melatonin attenuates EBI following SAH, by inhibiting the NLRP3 inflammasome and following neuroinflammation
Feng et al. (2015)	ICH	Vivo	Rats	P2X7R/NLRP3/ IL-1β(18)	P2X7R/NLRP3 axis inhibition reduces neuroinflammation after ICH
Yang et al. (2015)	ICH	Vitro/Vivo	Mice/Primary neuron culture	miR-223/ NLRP3/Caspase-1/ IL-1β	miR-223-mediated NLRP3 participated inflammation through caspase-1 and IL-1β.
Weng et al. (2015)	ICH	Vitro/Vivo	Mice/N9 microglial cell lines	NMDAR1/NLRP3/IL-1β	Elevated NMDAR1 expression and NMDAR1 phosphorylation may subsequently activate NLRP3 and IL-1β after ICH
Lin et al. (2018)	ICH	Vivo	Mice	Caspase-1/IL-1β	AC-YVAD- CMK could reduce caspase-1 activation and inhibit IL-1β production and maturation
Chen et al. (2019)	ICH	Vivo	Mice	MC4R/ASK1/JNK/p38 MAPK	RO27-3225 improved neurological functions and inhibited NLRP1-dependent neuronal pyroptosis *via* mediating MC4R/ASK1/JNK/p38 MAPK signaling pathways after ICH

The NLRP1 inflammasome is the first member that has been characterized among the NLR family. It has been reported to be primarily expressed in neurons and glial cells (Abulafia et al., 2009; Tan et al., 2014). The melanocortin 4 receptor (MC4R) is a seven-transmembrane G-protein–coupled receptor that could be activated by neuropeptide α-MSH, which exerts anti-inflammatory and neuroprotective effects after traumatic brain injury and cerebral ischemia (Forslin Aronsson et al., 2006; Yang et al., 2014). Treatment with the MC4R agonist, RO27-3225, successfully attenuated NLRP1-dependent neuron pyroptosis (including the cleaved caspase 1 and IL-1β level) after ICH in mice (Chen et al., 2019). Furthermore, RO27-3225 also reduced the expression of p-ASK1, JNK, and p-p38 mitogen-activated protein kinase (p38 MAPK) after ICH. This study presented a hypothesis that RO27-3225–mediated neuronal pyroptosis suppression may be regulated by the activation of MC4R and the inhibition of ASK1/JNK/p38 MAPK signaling pathways (Chen et al., 2019).

NLRP3 is another member of the NLR family reported in the central nervous system (CNS), with expression primarily located within the microglia and endothelium (Ma et al., 2014; Yang et al., 2014; Lin et al., 2018). Previous studies investigated several upstream regulators of NLRP3 involved in ICH pathophysiology. The ATP-gated transmembrane cation channel purinergic 2X7 receptor (P2X7R) is the key regulator in upstream activation of the NLRP3 inflammasome (Di Virgilio, 2007). P2X7R was activated after ICH and SAH *in* *vivo*. Inhibition of P2X7R activity can reduce NLRP3, IL-1β, and IL-18 from microglia (Chen et al., 2013; Feng et al., 2015). It was also postulated that peroxynitrite (ONOO^−^) could be involved in the P2X7R-regulated NLRP3 inflammasome formation after ICH (Feng et al., 2015).

Another study found evidence that microRNA-223 was a potential negative regulator of NLRP3 formation by using TargetScan (a searching program for potential regulator microRNA; Yang et al., 2015). The NLRP3 mRNA has conserved miR-223 binding sites in its 3′ UTR, which could be used to regulate the expression of NLRP3. Inhibition of NLRP3 by miR-223 reduces erythrocyte lysis-induced microglial inflammation and neuronal injury after ICH in mice (Yang et al., 2015). It was also found that the miR-223 levels decreased after erythrocyte lysis stimulation was decreased in microglia and ICH in mice (Yang et al., 2015). Meanwhile, *N*-methyl-D-aspartic acid receptor 1 (NMDAR1) was also suggested to play an important role in NLRP3 regulation after ICH *in vivo* and *in vitro* (Weng et al., 2015). The expression and phosphorylation of NMDAR1 were significantly increased after ICH and in cultured microglial cells treated with hemin (Weng et al., 2015). Furthermore, they demonstrated that an NMDAR1 inhibitor (MK801) attenuated hemin-induced activation of microglia, subsequently leading to a decrease in NLRP3 and Il- 1β microglia production (Weng et al., 2015).

Although several studies have attempted to investigate the expression of inflammasomes and upstream regulators of canonical inflammasomes after ICH, few have explored the key protein, GSDMD, downstream of the inflammasome and the noncanonical inflammasome. As we know, pyroptosis is a GSDMD-related cell death process, where two inflammasome signaling pathways converge at the GSDMD and are executed following pore formation (Jia et al., 2019). In addition to the canonical-inflammasome activation, caspase 4/5/11 also participates in IL-1/IL-18 processing and cell death (Jia et al., 2019). We propose that future studies should investigate the role that GSDMD and caspase 4/5/11 signaling pathways have in hemorrhagic stroke.

## Mechanisms of BBB Dysfunction Secondary to Hemorrhagic Stroke

**Figure 4:**
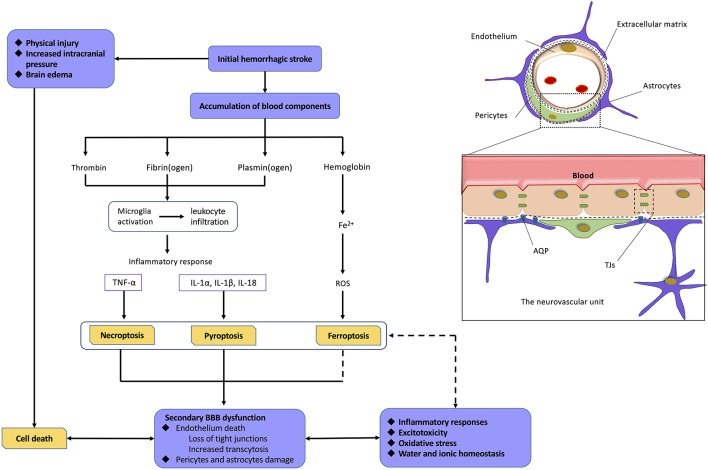
Relationship among hemorrhagic stroke, programmed cell deaths, and blood–brain barrier (BBB) dysfunction. Normal neurovascular units (NVUs) are composed of intact endothelial cells (ECs), pericytes, astrocytic endfeet, and extracellular matrix (ECM) components. ECs, with their tight junctions (TJs), are the most important components. On the one hand, initial hemorrhagic stroke directly causes cell death in central nervous system (CNS) as a result of physical injury, intracranial pressure, and brain edema. On the other hand, abundant blood components can also mediate programmed cell deaths (PCDs) *via* proinflammatory response, reactive oxygen species (ROS), and so on. The death of cell, especially ECs, leads to severe damage on BBB. As a result, BBB dysfunction induces a variety of pathophysiological processes including inflammatory responses, excitotoxicity, oxidative stress, water, and ionic homeostasis, which finally mediate more extensive PCDs. Abbreviations: TNFα, tumor necrosis factor; IL-1α/1β/18, interleukin 1α/1β/18.

The mechanism of BBB dysfunction after hemorrhagic is a complex process as shown in Figure 4. ICH and SAH are two categories of hemorrhagic stroke that occur in different intracerebral regions. ICH is mainly secondary to arteriolosclerosis induced by hypertension, coagulopathy, and vascular malformation rupture, whereas SAH is mainly caused by the rupture of an intracranial aneurysm (Gross et al., 2019; Zhang et al., 2019) Even with different etiologies, ICH and SAH are initially caused by vascular disruption in different intracerebral sites, which can be defined as the first phase of BBB disruption. This initial hemorrhage may result in early phases of cell death because of physical injury, brain edema, and increased intracranial pressure. At this stage, the death of cells comprising the NVU may significantly contribute to the induction of secondary BBB dysfunction (Hawkins and Davis, 2005; Knowland et al., 2014; Zhang et al., 2019). Currently, a normal BBB is no longer just a physical “barrier,” but a dynamic and metabolic interface, based on the novel structural foundation known as the NVU (Tso and Macdonald, 2014; Zou et al., 2017). This structure includes components, such as ECs and their linking TJs, pericytes, astrocytic end-feet, neurons, and extracellular matrix (ECM) components (Tso and Macdonald, 2014; Erdo and Krajcsi, 2019). These components interact with each other to form a highly connected entirety, which has a critical function in regulating the homeostasis of water and electrolytes, immune cell trafficking, transporting necessary nutrients, and preventing the entry of compounds into the brain (Abbott et al., 2010; Hladky and Barrand, 2016; Erdo and Krajcsi, 2019).

After hemorrhagic stroke, the blood components initiate the process of cell excitotoxicity, cell edema, oxidative stress, and neuroinflammation, which further disrupt the BBB. These neurotoxic blood–derived factors include thrombin, fibrin, and erythrocyte components (Keep et al., 2014; Tso and Macdonald, 2014; Zhu et al., 2019). Thrombin is the cascade product of prothrombin during hemostasis after hemorrhagic stroke. The binding of thrombin to protease activated receptor 1 induces secondary BBB disruption through phosphorylating Src kinases and activating microglia (Möller et al., 2006; Liu et al., 2010). Fibrin cleaved from fibrinogen has been reported to play a potential role in neuroinflammation and microglial activation (Lim-Hing and Rincon, 2017). Iron, degraded from hemoglobin, is one of most important factors among various components for hemorrhagic stroke–induced BBB hyperpermeability (Hua et al., 2007; Gomes et al., 2014), which is supported by ICH/SAH studies revealing alleviated brain edema with administration of the iron chelator, deferoxamine (Lee et al., 2010; Okauchi et al., 2010; Yu et al., 2014), or a heme oxygenase (HO) inhibitor (Wagner et al., 2000; Han et al., 2018). HO plays a critical role in the degradation of heme and the release of free ferrous iron (Kamat et al., 2019). Importantly, iron has been shown to mediate BBB dysfunction mainly through generating ROS, which can directly induce degradation of the endothelium and activate signaling pathways (Mori et al., 2001; Fraser, 2011). Meanwhile, the influx of albumin accompanied by disequilibrium of water and ion homeostasis leads to cerebral edema (Lehmann et al., 2013; Di Napoli et al., 2018).

It is widely accepted that the inflammatory response occurs when resident microglia of the brain are activated within the early stages of hemorrhagic stroke. This is due to stimuli, such as neurotoxic factor accumulation and reduced blood flow, which then causes the release of proinflammatory cytokines (Ge et al., 2018; Chen et al., 2019). Among other CNS diseases, such as cerebral ischemia, Alzheimer disease, or multiple sclerosis, the mechanisms of leukocytic infiltration into the brain and the immune response with the complement system have been widely studied (Jiang et al., 2018; Sweeney et al., 2018; Ma et al., 2019; Shen et al., 2019). DL-3-n-Butylphthalide, a synthetic compound that has been approved for the treatment of ischemic stroke in China, has been shown to inhibit neurovascular inflammation *via* downregulation of intercellular adhesion molecule 1 (ICAM-1; Yang et al., 2019). Despite a slight lag, it was recently revealed that these cytokines may also upregulate adhesion molecules, including ICAM-1, vascular adhesion molecule 1, and vascular adhesion protein 1 after hemorrhagic stroke (Ma et al., 2011; Xu et al., 2015; Cheng et al., 2018; Gris et al., 2019). These recruited leukocytes further accumulate and transmigrate across the endothelium, finally releasing an abundance of cytokines and chemokines (Cheng et al., 2018; Gris et al., 2019). Cytokines mediating various signaling pathways are an important part of the inflammatory processes after hemorrhagic stroke. For example, TNFα and IL-1β are both found to mediate cell function, including endothelial cell death, followed by BBB hyperpermeability (King et al., 2013; Yang et al., 2014; Liu et al., 2019). Chemokines, such as monocyte chemoattractant protein 1, not only can mediate leukocyte chemotaxis, but also can directly damage TJs. These both result in BBB dysfunction (Niwa et al., 2016; Yang et al., 2016). Meanwhile, more recent studies have focused on the immune response, with an emphasis on lymphocytic infiltration, as they contribute to brain recovery through hematoma resolution, neurogenesis, and axonal regeneration after ICH. Regulatory T cells were shown to mediate anti-inflammatory responses and improve neurological function in a chronic subdural hematoma rat model (Frost et al., 2019; Quan et al., 2019). These studies may provide emerging therapeutic targets for ICH (Shao A. et al., 2019). It should be noted that MMPs, which are primarily released by neutrophils, are also thoroughly studied potent proteinases and play a critical role in the increase of endothelial permeability *via* disruption of TJs, ECM degradation, and increased transcytosis of the endothelium (Abilleira et al., 2003; Qi et al., 2016; Liu et al., 2019). Recently, MMP-9 was also found to mediate endothelial permeability through the activity of von Willebrand factor and the initial recruitment of inflammatory leukocytes (Askenase and Sansing, 2016).

## Crosstalk Between BBB Dysfunction and PCDs in Hemorrhagic Stroke

Cell death, which occurs over an extended period following hemorrhagic stroke, not only can be directly induced by physical injury, but also indirectly mediated by pathophysiological process associated with BBB dysfunction mentioned above (Keep et al., 2014). The death of NVU cells further exacerbates BBB damage. While the three PCDs (necroptosis, ferroptosis, and pyroptosis) have distinct morphological, biochemical, and genetic characteristics, they have been shown to mutually participate in BBB dysfunction. The endothelium remains the primary target of study because of their paramount significance in the BBB (Figure 4; Zille et al., 2017; Chen et al., 2018, 2019; Zhao et al., 2018).

A significant amount of proinflammatory cytokines are increased during these three processes, such as TNFα, IL-1β, and IL-6 (Ge et al., 2018; Zhang Z. et al., 2018; Chen et al., 2019). As described above, upregulation of these cytokines will significantly induce microglial activation and increase BBB permeability, either directly or indirectly. To support this, several animal experiments have demonstrated this hyperpermeability of BBB *via* albumin extravasation or an Evans blue dye extravasation assay, and this pathological change can be reversed by specific inhibitors of necroptosis, ferroptosis, and pyroptosis (Chen et al., 2017; Ge et al., 2018; Zhang Z. et al., 2018; Yuan et al., 2019). More recently, research found that necroptosis occurred in the endothelium and is initiated by interactions between TNFα secreted by M1-type microglia and TNFR1 on the endothelium (Chen et al., 2019). The significant decrease in the levels of occludin and claudin-5 (major transmembrane TJs) and the increase in MMP-9 levels and activity can both be reversed by nec-1 after SAH (Chen et al., 2019). It implies that there is a high degree of correlation between necroptosis and BBB dysfunction after SAH. Similarly, pyroptosis of brain microvascular ECs was shown in a TBI model and was induced by GSDMD. BBB function and neurological outcomes improved when the process of pyroptosis was inhibited (Chen et al., 2018). According to the discovery above, it seems reasonable to speculate that ferroptosis also occurred in the endothelium after stroke events. In an ICH model, GPX4 reduced oxidative stress and thus inhibited ferroptosis to recover the function of the BBB (Zhang Z. et al., 2018). The inflammatory and innate immune responses are among the most important factors in BBB regulation. Recently, a series of experiments on aged mice revealed severe dysregulation of immunity and an excessive inflammatory response (Freitas et al., 2019; Shen et al., 2019; Zhang et al., 2019). As hemorrhagic stroke is a disease with an increased incidence in elderly populations, we should further investigate the effect of these changes on BBB dysfunction and PCDs.

Currently, research pertaining to ferroptosis and BBB dysfunction is very limited. However, according to the mechanisms of BBB dysfunction and ferroptosis discussed above, the role of ferroptosis on BBB damage warrants more attention. We have mentioned above with sufficient evidence that iron, when degraded from hemoglobin, has a critically important role in BBB hyperpermeability after hemorrhagic stroke, and decreasing iron content through various methods significantly reversed the brain edema (Wagner et al., 2000; Hua et al., 2007; Gomes et al., 2014; Yu et al., 2014). The key point of this problem lies in the poor understanding regarding the exact mechanism of iron overload-induced BBB hyperpermeability. Oxidative stress seems to be a promising candidate (Fraser, 2011). Interestingly, the mechanism of ferroptosis has not been fully clarified. However, iron and lipid peroxidation products have been demonstrated to be indispensable initiators of critical death signals (Yang and Stockwell, 2016; Stockwell et al., 2017). Moreover, studies had shown increased deleterious effects of lipid peroxidation products, such as HO-1, on free radical scavenging after ICH (Chang et al., 2014). Therefore, there are theoretical bases to prove the role of ferroptosis in BBB dysfunction.

In addition to the mechanisms of endothelial cell destruction being elucidated to some extent, these three PCDs may have some potential mechanisms relevant to other BBB structures and signaling pathways based on current research findings. Astrocytes are an important component of the BBB basic structure; they regulate electrolyte and water balance *via* an abundance of AQP-4 and gap junctions in the end-feet (Zlokovic, 2008). In the condition of depleted intracellular GSH, astrocytes undergo caspase-independent cell death. This process is highly relevant to the secretion of TNFα from human astrocytes and can be inhibited by nec-1 (Laird et al., 2008). It suggests that the studies regarding the exact mechanisms of necroptosis and nec-1 in astrocytes, as well as other BBB structures, strengthen the understanding of BBB dysfunction after hemorrhagic stroke.

Interestingly, there may also be some intersection between the three PCDs. Inducible GPX4 alleviates ferroptosis and pyroptosis in bacterial infection, whereas degradation of GSH enhances necroptosis and ferroptosis in human triple-negative breast cancer cells, suggesting a potential pathway involving pyroptosis and necroptosis (Zille et al., 2017; Zhu et al., 2019). Lastly, these three patterns of death may share common causes, which are not limited to oxidative stress. A logical plan of action would be to find shared targets of different PCDs to inhibit, therefore improving the efficacy of neurological recovery after hemorrhagic stroke.

## Conclusion

A growing number of studies show that PCDs are involved in hemorrhagic stroke. This review article summarizes the role of necroptosis, ferroptosis, and pyroptosis after hemorrhagic stroke, as well as their relationship with BBB dysfunction. Although most consequences of BBB dysfunction are detrimental, one potential beneficial result is that the entire brain would benefit if a novel therapeutic target was uncovered. Further studies exploring the mechanisms of these three forms of cell death and the crosstalk with BBB dysfunction will assist in the search for promising interventional targets of hemorrhagic stroke. Furthermore, more studies should broaden their scope to include research on cell types other than neurons.

## Author Contributions

All the authors participated in analyzing and discussing the literature, commenting on, and approving the manuscript. AS and JZ supervised the research, led the discussion, wrote and revised the manuscript. All authors read and approved the final manuscript.

## Conflict of Interest

The authors declare that the research was conducted in the absence of any commercial or financial relationships that could be construed as a potential conflict of interest.
